# Thoracoscopic versus open resection for symptomatic congenital pulmonary airway malformations in neonates: a decade-long retrospective study

**DOI:** 10.1186/s12890-021-01445-2

**Published:** 2021-03-12

**Authors:** Jintao Zheng, Huajian Tang, Huiyu Xu, Jiequan Li, Xiangming Mao, Guoqing Liu

**Affiliations:** 1 Department of Neonatal and Pediatric Surgery, Chancheng District, Foshan Women and Children Hospital Affiliated to Southern Medical University, No. 11 West Renmin Rd, Foshan City, 528000 Guangdong China; 2 Zhujiang Hospital, Southern Medical University, Guangzhou, 510282 Guangdong China

**Keywords:** Thoracoscopic resection, Congenital pulmonary airway malformations, Neonate

## Abstract

**Purpose:**

The purpose of this study is to evaluate the potential advantages of thoracoscopic versus open resection for symptomatic congenital pulmonary airway malformation (CPAM) in neonates.

**Methods:**

A retrospective review of the medical records of neonates (age ≤ 28 days) who underwent surgery for symptomatic CPAM from 2010 to 2020.

**Results:**

Of the 24 patients, 14 patients underwent thoracoscopic resection and 10 patients underwent open resection. 4 patients with CPAM located in the upper or middle lobes underwent lobectomy, and 20 underwent lung-preserving wedge resection in the lower lobe. Between the two groups, there were no statistically significant differences in related preoperative variables, including gestational age at birth, body weight, head circumference, lesion size, cystic adenomatoid malformation volume ratio (CVR), and age at operation (P > .05). The differences in intraoperative variables were statistically significant. The length of the surgical incision was significantly shorter in thoracoscopic resection group than in open resection group (1.4 cm [1.3–1.8] vs. 6.0 cm [5.0–8.0], P = .000), along with significantly less operative blood loss (3 ml [1–6] vs. 5 ml [2–10], P = .030) but significantly longer operation time (159 min [100–220] vs. 110 min [70–170], P = .003). Regarding postoperative variables, ventilator days, duration of chest tube use and length of hospital stay were not statistically significant (P > .05).

**Conclusion:**

Both thoracoscopic and open resection for symptomatic CPAM achieve good clinical outcomes, even in neonates. Thoracoscopic resection has minimal aesthetic effects and does not increase the risk of surgical or postoperative complications. Lung-preserving resection may be feasible for neonatal CPAM surgery.

## Introduction

Congenital pulmonary airway malformation (CPAM) is a foetal lung malformation characterized by benign malignancies or dysplastic lung tumours with excessive growth of terminal bronchioles and a decrease in the number of alveoli [1]. The reported incidence of CPAM is 1:11,000 to 1:35,000 [2]. The perinatal mortality rate associated with prenatally diagnosed CPAM varies widely, with a range of 9–49% [3], because the clinical characteristics of CPAM vary greatly, from acute respiratory distress at birth to incidental asymptomatic lesions on a chest radiograph at any age. Whether asymptomatic CPAM requires surgery during the neonatal period is still controversial, but it is clear that surgery is the accepted standard of care for all symptomatic CPAMs, even in neonates [4, 5]. Pneumonia, respiratory distress and shortness of breath are the common presenting symptoms [6]. We carried out surgical treatment for symptomatic CPAM patients after the above symptoms had adequately subsided. CPAM can be treated with open or thoracoscopic resection. To date, few reports have compared the outcomes of the two surgical methods for the treatment of CPAM in neonates. Therefore, the purpose of this study is to compare the preoperative, intraoperative, and postoperative variables of the two surgical procedures and to evaluate the potential advantages of thoracoscopic versus open resection for symptomatic CPAM in neonates.

## Methods

The study design was a retrospective review of the data from 24 symptomatic neonates (age ≤ 28 days) with CPAM who underwent thoracoscopic or open resection at the Foshan Women and Children Hospital Affiliated to Southern Medical University, from April 2010 to April 2020. The pathological results of all cases were confirmed to be CPAM or CPAM combined with pulmonary sequestration (PS). The medical records were reviewed for preoperative, intraoperative and postoperative variables. Preoperative variables included gestational age at birth, sex, body weight, head circumference, location of the CPAM, lesion size, cystic adenomatoid malformation volume ratio (CVR) [7], respiratory symptoms and age at operation. Intraoperative variables included length of surgical incision, operation time and operative blood loss. Postoperative variables included postoperative ventilator days, duration of chest tube use, length of postoperative hospital stay and postoperative complications.

**Fig. 1:**
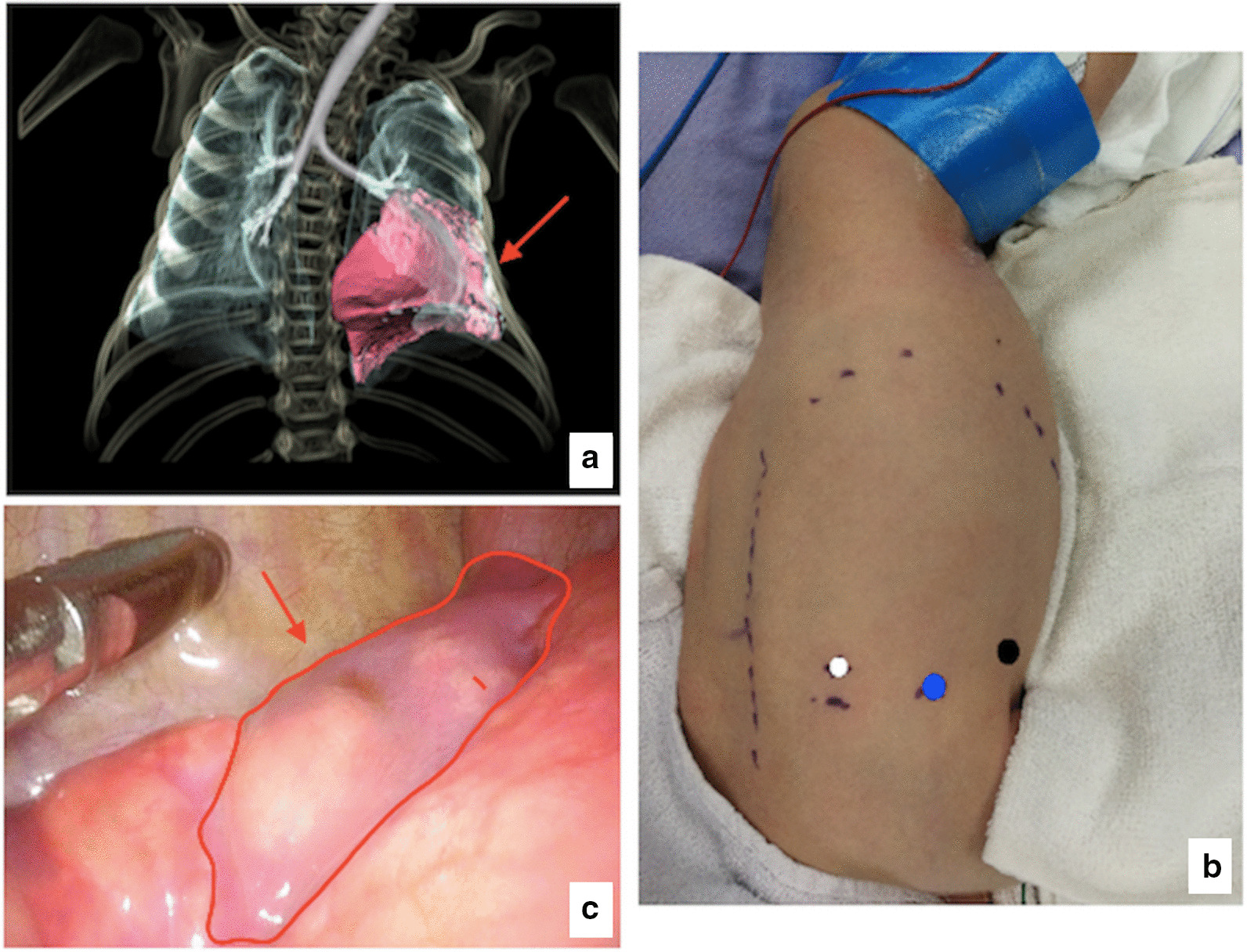
**a** Preoperative CT scan of a left lower lung segmental CPAM (arrows), cross-sectional view. **b** The patient was placed in a semiprone position with the affected side elevated as recommended for left thoracoscopic lower lesionectomy in a neonate (3-mm ports, white circles; a 5-mm port, blue circle, for camera; 5-mm port, black circle, for ultrasonically activated scalpel). **c** Intraoperative photograph of CPAM (arrows)

All cases were performed with preoperative CT or MRI scans and 3D reconstruction to better determine the range of the lesion (Fig. 1a). Surgery was performed after the symptoms had adequately subsided. Central venous and bladder catheters were placed.

For the thoracoscopic resection procedure, general anaesthesia, tracheal intubation and nonsingle lung ventilation were used. The patient was placed in a semiprone position with the affected side elevated to more easily expose the lesion. The first 5-mm trocar for thoracoscopy (5-mm 300, Olympus) was placed at the tip of the scapula. The second 5-mm trocar was inserted at the posterior axillary line, and the third 3-mm trocar was inserted at the interscapular region. Both trocars were parallel to the tip of the scapula (Fig. 1b). All trocars were sutured and fixed to prevent them from being pulled out. Carbon dioxide pressure was 3–5 mmHg. Thoracoscopic lobectomy or wedge resection was performed according to the size of the lesion (Fig. 1c). An ultrasonically activated scalpel was used for wedge resection, and the lung wound was sutured with 4–0 absorbable thread. Hemo-locks were used to clip the main blood vessel and main bronchus during lobectomy. The specimens were extracted in small pieces. The chest tube was conventionally placed.

Statistical analyses were performed using SPSS Statistics (version 12.0.0.0). The Mann–Whitney U test was used to compare continuous variables with nonparametric variables. Fisher’s exact test and 2 × 2 contingency tables were used to compare categorical variables. Statistical significance was defined as a P value of less than 0.05. This study was approved by the Medical Ethics Committee of Foshan Women and Children Hospital Affiliated to Southern Medical University, (FSFY-MEC-2020-031).

## Results

Twenty-four neonates with symptomatic CPAM underwent thoracoscopic resection or open resection at the Affiliated Foshan Maternity & Child Healthcare Hospital, Southern Medical University, from April 2010 to April 2020. Ten patients underwent open resection before April 2017; after that time, we became skilled in thoracoscopy technology, and the remaining 14 patients underwent thoracoscopic resection.

### Patient population and characteristics

Of the 24 patients, 14 were male and 10 were female. Twenty-three patients were diagnosed with prenatal sonography. One patient was diagnosed by chest radiograph due to shortness of breath after birth. The most common respiratory symptoms were pneumonia, respiratory distress and shortness of breath. All of the patients in this study had suffered from neonatal pneumonia; two patients had pneumonia with respiratory distress, and eight had pneumonia with shortness of breath. Two patients had mediastinal deviation due to large lung lesions. No case requires emergency surgery because of the above symptoms. Four patients with CPAM located in the upper or middle lobes underwent lobectomy, and 20 underwent lung-preserving wedge resection in the lower lobe, regardless of whether the lesion was on the left or right side. The final pathological result was 20 cases of CPAM and 4 cases of CPAM with PS. One patient had postoperative complications of an air leaks and healed spontaneously after conservative treatment. No thoracoscopic cases required conversion to open thoracotomy. The patients in this study had no postoperative bleeding, infection, pleural effusion or death. Details of the patient population and the characteristics of the thoracoscopic resection and open resection groups are shown in Table 1. Some variables were statistically analysed, and there was no statistically significant difference between the two groups.

**Table 1:** Patient population and characteristics

	Thoracoscopic resection (N = 14)	Open resection (N = 10)	P^*^
Sex (male/female)	7:7	7:3	.421
Prenatal diagnosis	13	10	1
Respiratory symptoms			
Preoperative pneumonia	14	10	–
Respiratory distress	1	1	1
Shortness of breath	3	5	.204
Mediastinal deviation	1	1	1
Resection method (lobectomy/lung-preserving)	2:12	2:8	1
Laterality (left-sided/right-sided)	9:5	4:6	.408
Location of the CPAM	Upper 2, lower 12	Upper 1, middle 1, lower 9	–
Pathology	10 CPAM, 4 CPAM with PS	10 CPAM	–
Postoperative complications	1 air leaks	0	1

^*^, P values refer to Fisher’s exact test. CPAM, congenital pulmonary airway malformation; PS, pulmonary sequestration

All cases were followed up continuously. The median follow-up time was 25 months (range 1–47 months), and none of the patients experienced shortness of breath, difficulty breathing after activity, repeated pneumonia or developmental retardation of the nervous system. We perform CT examinations for those patients after surgery at 12 months. To date, twenty patients have undergone CT examination, and no postoperative recurrence has occurred. One patient who was treated with open resection had a slightly sunken chest while breathing.

### Results of the comparison of related variables: thoracoscopic versus open resection

**Fig. 2:**
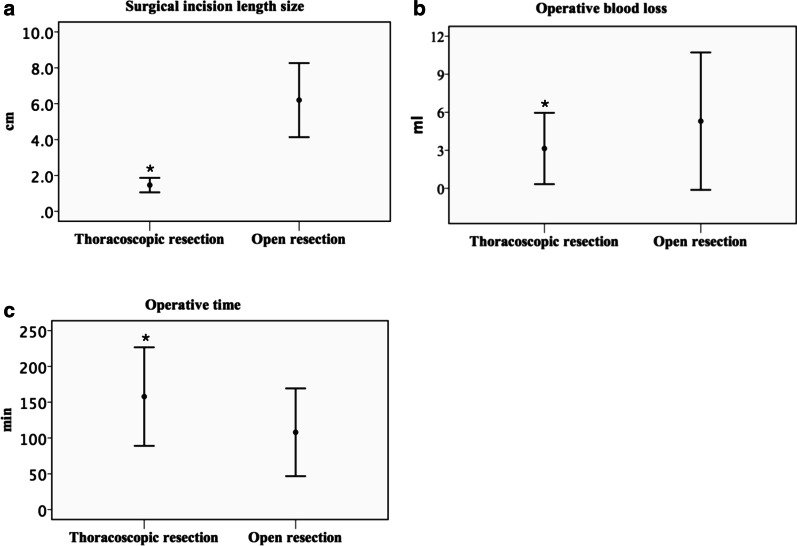
The differences in surgical incision length (P = .000) (**a**), operative blood loss (P = .030) (**b**) and operation time (P = .003) (**c**) between the thoracoscopic resection group and the open resection group were statistically significant

There were no statistically significant differences between the thoracoscopic resection group and the open resection group in the related preoperative variables (median [range]), including gestational age at birth (39.4 w [34.1–41.1] vs 39.3 w [35.5–41.4], P = 0.977), body weight (3260 g [2100–4270] vs 3240 g [2760–3860], P = 0.838), head circumference (33 cm [32–37] vs 34 cm [31–34], P = 0.785), lesion size (24.00 cm^3^ [12.00–63.00] vs 34.85 cm^3^ [8.00–125.00], P = 0.319), CVR (0.37 [0.18–1.00] vs 0.55 [0.13–1.92], P = 0.266), and age at operation (10 d [5–21] vs 11 d [7–17], P = 0.442) (Table 2). The related intraoperative variables showed statistically significant differences between the groups. The length of the surgical incision in the thoracoscopic resection group was significantly shorter than that in the open resection group (1.4 cm [1.3–1.8] vs 6.0 cm [5.0–8.0], P = 0.000), and there was significantly less blood loss in the thoracoscopic resection group (3 ml [1–6] vs 5 ml [2–10], P = 0.030), but their operation time was significantly longer (159 min [100–220] vs 110 min [70–170], P = 0.003) (Fig. 2). Regarding the related postoperative variables, the postoperative ventilator days (3 d [1–6] vs 2 d [1–3], P = 0.159), duration of chest tube use (8 d [4–22] vs 6 d [4–15], P = 0.052) and length of postoperative hospital stay (12 d [10–25] vs 12.5 d [9–16], P = 0.425) did not significantly differ between the two groups.

**Table 2:** Comparison of related variables: thoracoscopic resection versus open resection

Related variables	Thoracoscopic resection (N = 14)	Open resection (N = 10)	P^*^
	Median (range)	Median (range)	
Preoperative			
Gestational age at birth (w)	39.4 (34.1–41.1)	39.3 (35.5–41.4)	.977
Body weight (g)	3260 (2100–4270)	3240 (2760–3860)	.838
Head circumference (cm)	33 (32–37)	34 (31–34)	.785
Lesion size (cm^3^)	24.00 (12.00–63.00)	34.85 (8.00–125.00)	.319
CVR	0.37 (0.18–1.00)	0.55 (0.13–1.92)	.266
Age at operation (days)	10 (5–21)	11 (7–17)	.442
Intraoperative			
Surgical incision length (cm)	1.4 (1.3–1.8)	6.0 (5.0–8.0)	.000
Operative blood loss (ml)	3 (1–6)	5 (2–10)	.030
Operation time (min)	159 (100–220)	110 (70–170)	.003
Postoperative			
Postoperative ventilator days (days)	3 (1–6)	2 (1–3)	.159
Duration of chest tube use (days)	8 (5–22)	6 (4–15)	.052
Length of postoperative hospital stay (days)	12 (10–25)	12.5 (9–16)	.425

^*^P values are based on the Mann–Whitney U test. w, weeks; g, grams; cm, centimetres; d, days; ml, millilitres; min, minutes; CVR, cystic adenomatoid malformation volume ratio

## Discussion

Congenital pulmonary airway malformation (CPAM) is a lung malformation in which bronchioles proliferate abnormally and form various sizes of cysts in the foetal period [1]. Most CPAMs are not associated with severe respiratory symptoms after birth, but approximately 10% of lung lesions, including CPAMs, will present with symptoms during the neonatal period [8]. Infection, shortness of breath, respiratory distress, and mediastinal deviation are common presenting symptom as indicated by the patients in this study. The optimal timing for the resection of asymptomatic CPAM remains controversial, but surgery is the accepted standard for all symptomatic CPAMs [9, 10], and thoracoscopic operations are safe and feasible, even in neonates [11, 12]. After the symptoms subsided sufficiently, we performed surgical treatment of symptomatic CPAM patients. Those clinical symptoms included curing of pneumonia and disappearance of the respiratory distress or shortness of breath without relying on supplemental oxygen. For asymptomatic CPAM patients, we choose to observe and follow up regularly.

This study shows that both thoracoscopic and open resection for symptomatic CPAM achieved good clinical outcomes in the neonatal period. When the two surgical methods are compared under the same preoperative conditions, thoracoscopic resection results in a shorter surgical incision and less operative blood loss, but the operation time is longer. Therefore, thoracoscopic surgery achieved an aesthetic effect consistent with minimally invasive surgery. These clinical results are similar to those described in the literature [4, 13, 14]. In our experience, neonatal CPAM has a clear boundary with normal lung tissue and no adhesions caused by repeated infections. The blood vessels and bronchial tubes are relatively small and can be directly cut off by an ultrasonically activated scalpel. These characteristics are conducive to thoracoscopic resection. Thoracoscopic resection of CPAM has one unfavourable factor: the operation space is small, making it difficult to perform this operation during the neonatal period. Prior to this, we performed thoracoscopic operations for congenital diaphragmatic hernia and extra-lobar pulmonary sequestration to improve our skills. In addition, when suturing is difficult, a 3-mm trocar can also be added to assist. If blood oxygen maintenance is unstable during the operation, it is necessary to temporarily stop the operation and stop the artificial pneumothorax.

The extent of surgical resection in the management of CPAM also remains controversial [15]. Muller et al. [16] state that adequate treatment of CPAM in children requires lobectomy because of poor sensitivity and very poor negative predictive value of preoperative CT for determining distal adjacent lesions. Laberge et al. [6] recommend lobectomy in order to prevent postoperative air leaks and residual disease and perhaps reduce the risk of some later malignancies. Fascetti-Leon et al. [17] state that lung-sparing surgery for congenital lung malformations is a safe and effective method of lung parenchymal preservation in paediatric patients. If accurately planned in selected patients, lung-sparing surgery does not carry a higher risk of residual disease and recurrence than traditional lobectomy. Kim et al. [18] report that early and late outcomes are excellent even after parenchyma-saving resection in patients with CPAM and suggest that parenchyma-saving resection can be safely performed in selected patients with a well-confined CPAM lesion, thereby avoiding lobectomy.

In this study, twenty patients underwent lung-preserving wedge resection for the treatment of CPAM of the lower lobe and achieved good clinical outcomes. Instead of ligating the bronchus, the lung wound was sutured with 4–0 absorbable thread in order to avoid pneumothorax caused by non-healing of the wound, and based on the literature [13], we extended the time of duration of chest tube use accordingly. Four patients underwent lobectomy because the CPAM almost or completely occupied the upper or middle lobe, preventing the use of the lung-preserving strategy. One early patient had the postoperative complication of air leaks, which were cured by continuous thoracic drainage. We think that this complication was caused by poor wound healing rather than a problem with the lung preservation strategy because there was no recurrence in the postoperative follow-up. It may also have been related to unskilled suturing under thoracoscopy at an early stage of the procedure, but the complication rate was not statistically significantly different from that of open resection (P = 1).

## Conclusion

Based on the data and follow-up results of this study, we believe that both thoracoscopic and open resection for symptomatic CPAM achieve good clinical outcomes, even in neonates. Compared with open resection, thoracoscopic surgery has minimal aesthetic effects and does not increase the risk of surgical or postoperative complications. The lung-preserving resection strategy may be feasible for neonatal CPAM patients. However, the study results are limited, notably because of the small sample size. In the next step, we will continue to perform long-term follow-up and evaluate the respiratory function of these patients.

## Data Availability

The datasets used and/or analyzed during the current study are available from. the corresponding author on reasonable request.

## Abbreviations

CPAM: Congenital pulmonary airway malformation
PS: Pulmonary sequestration
